# Prognostic value of nutritional parameters in systolic heart failure with renal dysfunction

**DOI:** 10.1371/journal.pone.0266839

**Published:** 2022-05-19

**Authors:** Takahiro Doi, Takahiro Noto, Tomohiro Mita, Daigo Nagahara, Satoshi Yuda, Akiyoshi Hashimoto, Tomoaki Nakata, Kenichi Nakajima

**Affiliations:** 1 Department of Cardiology, Teine Kijinkai Hospital, Sapporo, Hokkaido, Japan; 2 Department of Cardiology, Renal and Metabolic Medicine, Sapporo Medical University, Sapporo, Japan; 3 Department of Cardiology, Hakodate Goryokaku Hospital, Hakodate, Japan; 4 Depeatment of of Functional Imaging and Artificial Intelligence, Kanazawa University, Kanazawa, Japan; Universita degli Studi di Napoli Federico II, ITALY

## Abstract

Although it is known that assessment and management of the nutritional status of patients are important for treatment of patients with heart failure (HF), there are currently no established indicators. Therefore, we investigated the effects of nutritional parameters as well as conventional parameters on the prognosis of HF patients. A total of 1954 consecutive HF patients with left ventricular ejection fraction (LVEF) less than 50% were enrolled in this study. Transthoracic echocardiography was performed and conventional parameters for HF patients and parameters to assess nutritional status were measured in all patients. Patients were followed up with a primary endpoint of lethal cardiac events (CEs) for 30.2 months. During the follow-up period, cardiac events were documented in 619 HF patients. The CEs group had a lower level of cholinesterase (201.5U/L vs 265.2U/L, P <0.0001), lower estimated GFR (35.2 ml/min/1.73m^2^ vs 50.3ml/min/1.73m^2^, P< 0.0001), and lower Geriatric Nutritional Risk Index (GNRI) (91.9 vs 100.0, P< 0.0001) than those in the non-CEs group. Serum cholinesterase, estimated GFR, and GNRI were identified as significant prognostic determinants in multivariate analysis. ROC analyses revealed cut-off values of serum cholinesterase, estimated GFR, and GNRI of 229U/L, 34.2 ml/min/1.73m^2^, and 95.6, respectively, for identifying high-risk HF patients. HF patients with serum cholinesterase< 229U/L, estimated GFR<34.3 ml/min/1.73m^2^, and GNRI< 95.6 had a significantly greater rate of CEs than that in the other patients (P<0.0001). Low serum cholinesterase and low GNRI can predict cardiac mortality risk in systolic HF patients with renal dysfunction.

## Introduction

The number of patients with heart failure (HF) is increasing worldwide due to aging of the population and increase in lifestyle-related diseases such as hypertension, diabetes mellitus, and dyslipidemia [1, 2]. Although advances in pharmacological and non-pharmacological therapies have steadily improved the life expectancy of patients with HF [3, 4], they are still inadequate and there is a need to develop and establish more effective and efficient treatment and management. The goals of heart failure treatment are not only to improve prognosis but also to reduce symptoms and improve exercise tolerance and quality of life. The more severe heart failure is, the more important it is to improve and maintain physical functions.

The importance of improving and maintaining physical functions increases as the severity of heart failure progresses [2]. It has been known for a long time that physical inactivity in patients with heart failure is caused by a wasting state called cachexia in severe cases, but in recent years, attention has been given to the involvement of sarcopenia and frailty [5]. Therefore, dietary therapy, especially nutritional therapy, is important as well as exercise therapy in the treatment of HF.

Progress has been made in both basic research and clinical research on exercise therapy for patients with HF. The usefulness of exercise therapy has been proven, and standardized methods based on the results of studies have been established. On the other hand, despite the importance of nutritional therapy for patients with HF, methods for assessing nutritional status and management based on these methods have not yet been established.

In recent years, nutritional parameters such as serum cholinesterase and Geriatric Nutritional Risk Index (GNRI) have been reevaluated as indicators of liver function and nutrition, and several studies have revealed their relationships with prognosis in patients with HF [6, 7]. However, there are few reports on the relationships of serum cholinesterase and GNRI with renal dysfunction and the correlations between these parameters and prognosis in HF patients.

This study demonstrated that serum cholinesterase and GNRI were useful as prognostic factors in systolic heart failure patients. Moreover, these parameters, when combined with a low eGFR as a surrogate for renal dysfunction, risk-stratify systolic heart failure patients synergistically.

## Materials and methods

### Study participants and data collection

The study was approved by the Ethics Committee of Obihiro Kousei Hospital, with approval number 2016–055.

A total of 1954 consecutive patients with symptomatic HF who were admitted to our hospital between April 2010 and December 2016 were enrolled in this retrospective study. The entry criteria were as follows: symptomatic HF, LVEF of less than 50% on echocardiography and age of 20 years or older. Patients with obvious malignancies, hematological or hepatic diseases, and bleeding disorders and patients under 20 years of age were excluded. The patients included 1464 males and 490 females. The mean age of the patients was 68.4±12.5 years and the mean value of LVEF and body mass index (BMI) were 33.8±10.8% and 22.6±4.5kg/m^2^, respectively. The basis for the diagnosis of decompensated HF was the Framingham criteria, which included typical symptoms, distended jugular veins, lung rale, peripheral edema, S3 or S4 gallop, and tachycardia. A chest X-ray, chest computer tomography and two-dimensional echocardiography were performed to support the diagnosis and to rule out other diseases with similar symptoms such as respiratory distress and chest discomfort and signs. In addition to a definitive history of prior myocardial infarction and/or coronary artery revascularization, HF etiologies such as ischemic or non-ischemic etiologies were also established by using a 12-lead electrocardiogram, exercise or drug-stress testing with or without cardiac imaging, and noninvasive or invasive coronary angiographic examination. Just before discharge, levels of hemoglobin (Hb), serum cholinesterase and creatinine, brain natriuretic peptide (BNP) and N-terminal pro-brain natriuretic peptide (NT-pro BNP) were measured. Total protein, albumin, and lipid profiles were measured simultaneously. Renal function was also evaluated by estimated glomerular filtration rate (eGFR) using the standard formula. Baseline GNRI was calculated from serum albumin and BMI using the following formula: GNRI = 14.89 × serum albumin (g/dL) + 41.7 × present body weight/[height2 (m2) × 22] [8, 9]. Plasma BNP levels were measured in 1312 patients (67.1%), while NT-pro BNP levels were measured alternatively in the remaining 642 patients (32.9%). BNP and NT-pro BNP were classified into four stages based on the ESC heart failure guidelines because BNP was assessed in two different parameters: BNP and NTproBNP were 0–40 pg/ml and 0–125 pg/ml for stage 1, 41–100 pg/ml and 126–400 pg/ml for stage 2, 101–200 pg/ml and 401–900 pg/ml for stage 3, and 201~ pg/ml and 901~ pg/ml for stage 4, respectively, and statistical analysis was performed.

Two-dimensional echocardiography was performed from parasternal long-axis and apical four-, three- and two-chamber views in a left lateral decubitus position using Vivid 7 or Vivid E9 (GE Healthcare Japan Co., Japan). The following echocardiographic parameters were measured in a compensated condition of HF prior to discharge: left atrium diameter (LAD; mm), left ventricular end-diastolic diameter (LVDd; mm), left ventricular ejection fraction (LVEF; %) calculated using the biplane modified Simpson’s method, left ventricular volume at end-diastole (EDV; ml), left ventricular volume at end-systole (ESV; ml) and septal E/e’. The laboratory technicians and echocardiographers were not informed of the results of this analysis.

### Follow-up protocol

The enrolled HF patients were retrospectively registered in our HF database and were followed up by cardiologists on a regular outpatient basis for more than one year, provided the patients were still alive. The primary endpoints used in this study were fatal cardiac events, including sudden cardiac death, death from progressive pump failure, and fatal ventricular tachyarrhythmias, as well as appropriate ICD therapy for these fatal arrhythmias. After confirming the clinical outcomes by reviewing the medical records, the following outcome analysis was conducted. Sudden cardiac death was defined as witnessed cardiac arrest and death within 1 hour of the onset of acute symptoms, or unexpected death in a patient who had been well within the previous 24 hours.

The present study was based on the principles of the Declaration of Helsinki and was conducted in accordance with the guidelines of the Ethics Committee of our hospital. Since this was a retrospective observational study, we informed the patients about the study by posting a notice on the hospital’s website. The ethics committee determined that we had obtained comprehensive consent from the patients, and the need for consent was waived.

### Statistical analysis

Statistical values are presented as means ± SD. The paired t-test was used to compare means between the two groups, and the chi-square test was used to compare categorical variables. Following univariate analysis, multivariate analysis with the Cox proportional hazards model was performed using a statistically appropriate number of significant variables that were identified in the univariate analysis dependent on the number of cardiac events (variables with p <0.15) and had previously been reported to be strongly correlated with prognosis of heart failure in order to calculate hazard ratios and 95% confidence intervals (CIs) for the significant variables. Receiver operating characteristic (ROC) analysis was also performed to determine the optimal cut-off values for the independent significant parameters. Kaplan-Meier analysis using the key parameters identified in this study was used to generate time-dependent cumulative event-free curves.

In this study, the computer software SAS for Windows, version 9.4 (SAS Institute, Cary, North Carolina, USA) was used for statistical analyses, and a p-value of less than 0.05 was considered significant. Moreover, Mathematica software (version 12.3, Wolfram Research Inc., Champaign, IL. USA) was used to test the complementary log-log plot to confirm proportional hazards [10, 11].

## Results

During a mean follow-up period of 30.2±18.4 months, cardiac events (CEs) were documented in 619 (31.6%) of the patients: HF death occurred in 492 patients due to progressive pump failure, lethal ventricular arrhythmias occurred in 34 patients, sudden cardiac death occurred in 71 patients and appropriate ICD shocks against lethal ventricular arrhythmias occurred in 22 patients. Patients with CEs were older than patients without CEs and had a greater NYHA functional class, lower eGFR (35.2±25.7 ml/min/1.73m^2^ vs 50.3±29.6 ml/min/1.73m^2^, *P<0*.*0001*), lower BMI (21.7±4.7 kg/m^2^ vs 23.1±4.4 kg/m^2^, *P<0*.*0001*), lower serum cholinesterase (201.5±82.3 U/L vs 265.2±84.3 U/L, *P<0*.*0001*), and lower GNRI (91.9±13.6 vs 100.0±12.9, *P<0*.*0001*) than those in patients without CEs (Table 1). Patients with CEs had larger left ventricular end-systolic volume (ESV) (112.3±57.3 ml vs 96.0±48.3 ml, *P<0*.*0001*) and greater septal E/e’ (19.0±8.4 vs 16.9±7.3 kg/m^2^, *P<0*.*0001*) than those in patients without CEs (Table 1).

**Table 1:** Comparison of clinical data and two-dimensional echocardiographic parameters in the groups with and without cardiac events.

	Cardiac events group (n = 619)	Non-cardiac events group (n = 1335)	p-value
**Age (years)**	72.1±11.0	66.7±12.9	P <0.0001
**Gender (male/female)**	450/169	1015/320	P = 0.2015
**NYHA classification (I~III/III~IV)**	472/177	1318/17	P < 0.0001
**BMI (kg/m** ^**2**^ **)**	21.4±4.	23.1±4.4	P < 0.0001
**Past history**			
**Hypertension**	261 (42.1%)	631 (47.2%)	P = 0.0291
**Diabetes mellitus**	211 (34.1%)	494 (37.0%)	P = 0.1937
**Dyslipidemia**	213 (34.4%)	574 (42.9%)	P = 0.0003
**Atrial fibrillation**	203 (32.7%)	401 (30.0%)	P = 0.1243
**Ventricular tachycardia/ ventricular fibrillation**	125 (20.2%)	180 (13.4%)	P = 0.0002
**Etiology**			
**Ischemic**	322 (52.0%)	667 (49.9%)	P = 0.4348
**Previous MI**	258 (41.7%)	497 (37.2%)	P = 0.0738
**Post PCI**	206 (33.3%)	492 (36.8%)	P = 0.1057
**Post CABG**	112 (18.1%)	208 (15.6%)	P = 0.1744
**Device implantation**			
**ICD implantation**	80 (12.9%)	155 (11.6%)	P = 0.4192
**CRT implantation**	56 (9.0%)	124 (9.3%)	P = 0.8513
**Laboratory data**			
**Hemoglobin (g/dL)**	11.4±2.2	12.5±2.2	P < 0.0001
**eGFR (ml/min/1.73m**^**2**^**)**	35.2±25.7	50.3±29.6	P < 0.0001
**Sodium (mmol/L)**	138.6±4.8	139.3±5.3	P < 0.0001
**BNP/NTproBNP staging (I/II/III/IV)**	15/36/56/512	94/182/193/866	P < 0.0001
**Serum cholinesterase (U/L)**	201.5±82.3	265.2±84.3	P < 0.0001
**Total cholesterol (mg/dl)**	161.3±40.5	170.1±38.3	P < 0.0001
**Low-density lipoprotein cholesterol (mg/dl)**	91.1±35.6	99.0±34.3	P < 0.0001
**High-density lipoprotein cholesterol (mg/dl)**	44.5±16.8	47.9±20.9	P < 0.0001
**Triglycerides (mg/dl)**	110.8±62.2	127.3±86.2	P < 0.0001
**Total protein (g/L)**	6.6±0.8	6.9±0.7	P < 0.0001
**Albumin (g/L)**	3.4±0.6	3.8±0.6	P < 0.0001
**Nutritional parameter**			
**Geriatric Nutritional Risk Index**	91.9±13.6	100.0±12.9	P < 0.0001
**Medication**			
**ACE-I/ARB**	354 (57.2%)	782 (58.6%)	P = 0.3465
**β-blockers**	574 (92.8%)	1245 (93.3%)	P = 0.2345
**Loop diuretics**	472 (76.3%)	1026 (76.9%)	P = 0.3375
**Mineralocorticoid receptor antagonist**	181 (29.3%)	408 (30.6%)	P = 0.2315
**Anti-vasopressin agents**	100 (16.3%)	180 (13.5%)	P = 0.0382
**Amiodarone**	262 (42.4%)	336 (25.2%)	P = 0.0068
**Statins**	221 (35.7%)	649 (48.6%)	P = 0.0849
**Findings of echocardiographic parameters**			
**M-mode**			
**LVDd (mm)**	55.7±8.7	54.6±10.8	P = 0.0204
**LVDs (mm)**	46.0±9.8	45.6±11.9	P = 0.4813
**LAD (mm)**	43.4±8.3	42.7±8.1	P = 0.0931
**Modified Simpson method**			
**LVEF (%)**	32.9±11.3	34.3±10.5	P = 0.0109
**EDV (ml)**	151.3±77.1	150.7±55.0	P = 0.8426
**ESV (ml)**	112.3±57.3	96.0±48.3	P < 0.0001
**Doppler method**			
**E wave velocity (m/sec)**	0.83±0.30	0.82±0.30	P = 0.9327
**Dct (msec)**	189.4±91.7	194.9±79.3	P = 0.1780
**Tissue Doppler method**			
**Septal E/e’**	19.0±8.4	16.9±7.3	P <0.0001

Values are shown as means±one standard deviation, MI, myocardial infarction; PCI, percutaneous coronary intervention; CABG, coronary artery bypass grafting; ICD, implantable cardioverter-defibrillator; CRT, Cardiac resynchronization therapy; eGFR, estimated glomerular filtration rate; NYHA, New York Heart Association; ACE-I, angiotensin-converting enzyme inhibitors; ARB, angiotensin receptor blockers;

LAD, left atrial diameter; LV, left ventricular; LVEF, left ventricular ejection fraction; LVDd, end-systolic left ventricular diameter; EDV, left ventricular end-diastolic volume; ESV, left ventricular end-systolic volume; Dct, deceleration time; ns, no significance.

In addition to the results of univariate analysis (Table 2), serum cholinesterase and GNRI as well as NYHA functional class and eGFR were confirmed to have significant independent prognostic values by multivariate Cox analysis with chi-square values of 24.3 (*P<0*.*0001*, hazard ratio: 0.996, 95% CI: 0.994–0.998) and 4.56 (*P = 0*.*0312*, hazard ratio: 0.990, 95% CI: 0.987–0.997), respectively.

**Table 2:** Results of univariate and multivariate analyses.

	**Univariate Analysis**				
			95% CI		
	χ^2^	Hazard ratio	Lower	Upper	p-value
**Age**	10.5	1.016	1.006	1.027	0.0012
**NYHA functional class ((I~II/III~IV))**	295	6.866	5.737	8.185	< 0.0001
**Atrial fibrillation**	1.10	1.089	0.926	1.267	0.2950
**Previous MI**	2.70	1.144	0.974	1.341	0.1002
**Hemoglobin**	9.86	0.919	0.871	0.969	0.0017
**Estimated GFR**	24.7	0.971	0.960	0.982	< 0.0001
**Sodium**	4.39	0.972	0.947	0.998	0.0360
**BNP/NTproBNP staging**	70.6	1.577	1.403	1.785	< 0.0001
**Serum cholinesterase**	228	0.991	0.990	0.993	< 0.0001
**Total cholesterol**	25.8	0.994	0.992	0.996	< 0.0001
**LDL cholesterol**	23.1	0.993	0.991	0.966	< 0.0001
**HDL cholesterol**	17.1	0.988	0.982	0.993	< 0.0001
**Total protein**	66.1	0.664	0.608	0.729	< 0.0001
**GNRI**	180	0.961	0.954	0.966	< 0.0001
**ESV**	7.14	1.001	1.000	1.002	0.0075
**LAD**	1.76	1.006	0.996	1.016	0.1835
**Septal E/e’**	41.8	1.034	1.025	1.045	< 0.0001
	**Multivariate Cox Proportional Hazards Model Analysis**				
			95% CI		
	χ^2^	Hazard ratio	Lower	Upper	p-value
**Age**	17.1	1.018	1.009	1.027	<0.0001
**NYHA functional class (I~III/III~IV)**	258	2.326	2.112	2.559	<0.0001
**Atrial fibrillation**	0.69	1.088	0.889	1.321	0.4054
**Previous MI**	1.02	1.099	0.915	1.318	0.3110
**Hemoglobin**	0.16	0.975	0.948	1.022	0.6891
**Estimated GFR**	14.7	0.993	0.988	0.996	0.0001
**Sodium**	2.70	0.985	0.973	1.003	0.1002
**BNP/NTproBNP staging**	1.90	1.091	0.923	1.256	0.1854
**Serum cholinesterase**	24.3	0.996	0.994	0.998	< 0.0001
**Total cholesterol**	0.01	1.000	0.996	1.004	0.9658
**LDL cholesterol**	1.26	1.002	0.996	1.006	0.2619
**HDL cholesterol**	0.07	0.999	0.992	1.004	0.7813
**Total protein**	2.10	0.911	0.807	1.032	0.1471
**GNRI**	4.56	0.990	0.987	0.997	0.0312
**ESV**	11.4	1.002	1.001	1.005	0.0007
**LAD**	0.03	1.001	0.988	1.014	0.8561
**Septal E/e’**	1.06	1.009	0.994	1.017	0.3018

**Fig 1:**
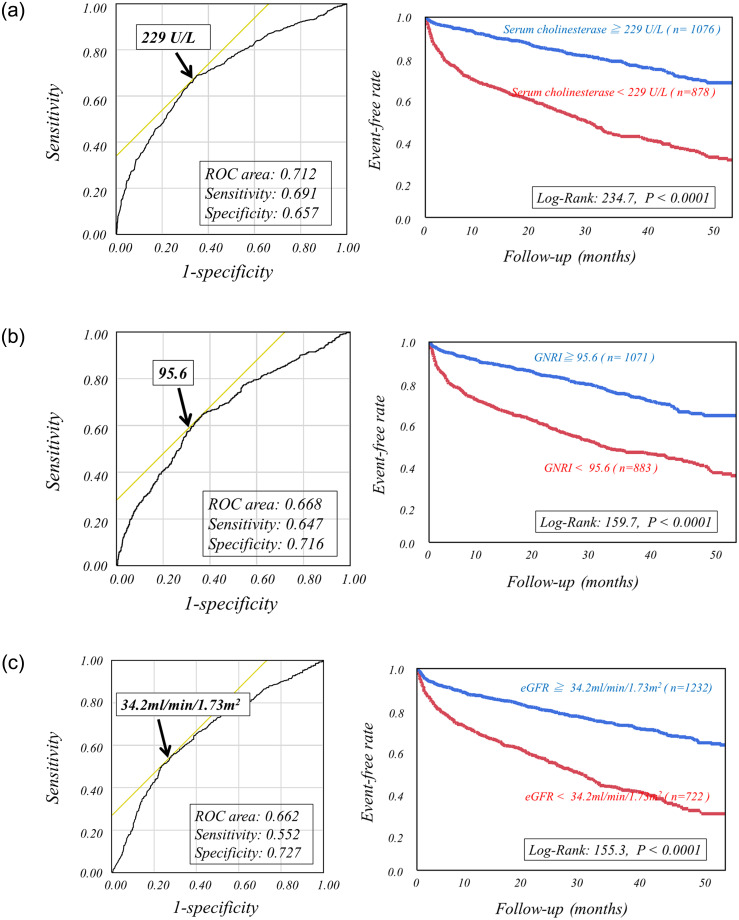
Kaplan-Meier event-free curves clearly discriminate high-risk patients from low-risk patients by using cutoff values determined by ROC analysis, including serum cholinesterase of 227U/L (A), Geriatric Nutritional Risk Index (GNRI) of 95.6 (B) and estimated GFR of 34.2ml/min/1.73m^2^ (C).

**Fig 2:**
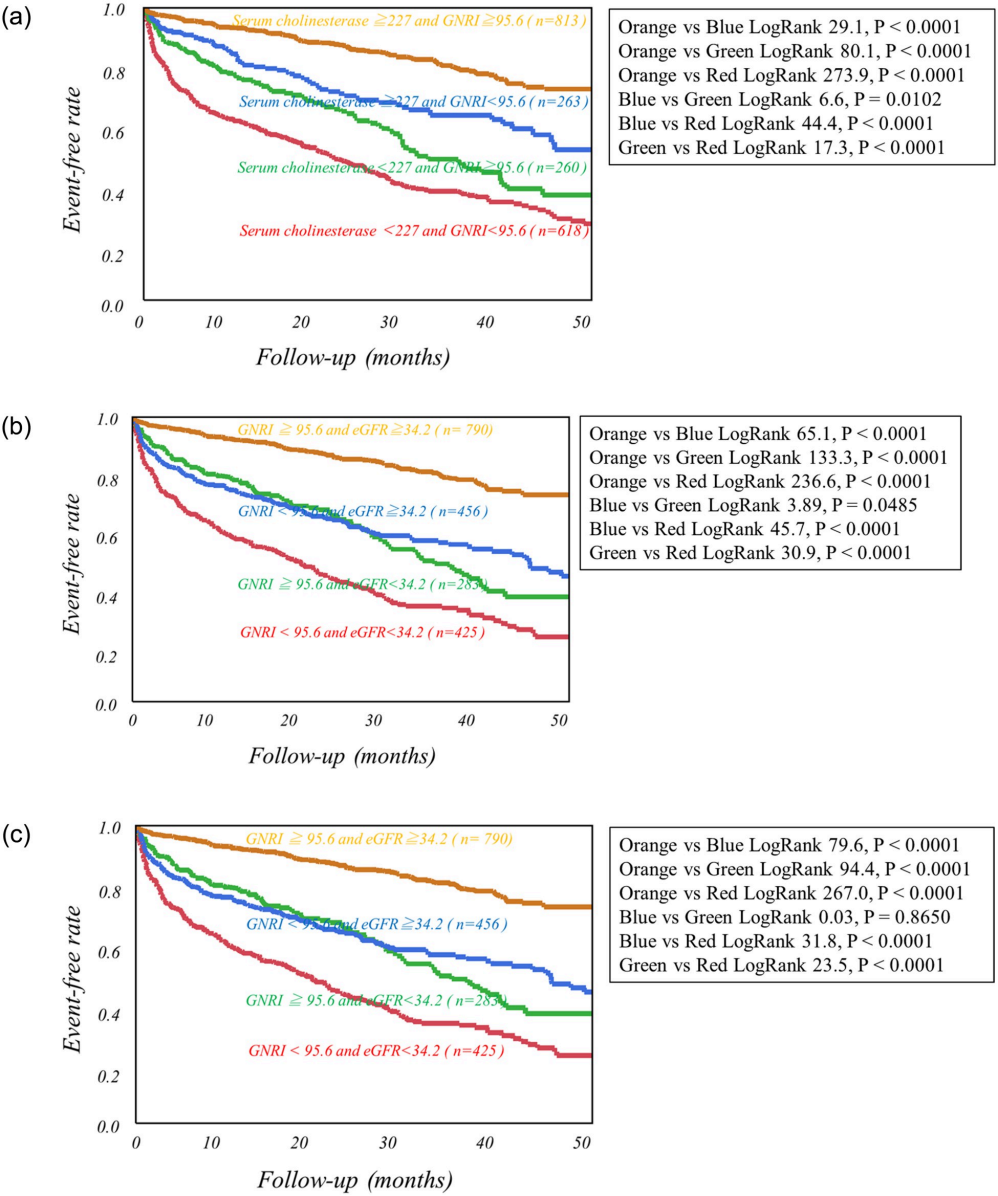
Kaplan-Meier event-free curves created by the combinations of two of the three prognostic variables including serum cholinesterase, Geriatric Nutritional Risk Index (GNRI) and estimated GFR. (a) Serum cholinesterase and Geriatric Nutritional Risk Index (b) Serum cholinesterase and estimated GFR (c) Geriatric Nutritional Risk Index and estimated GFR.

**Fig 3:**
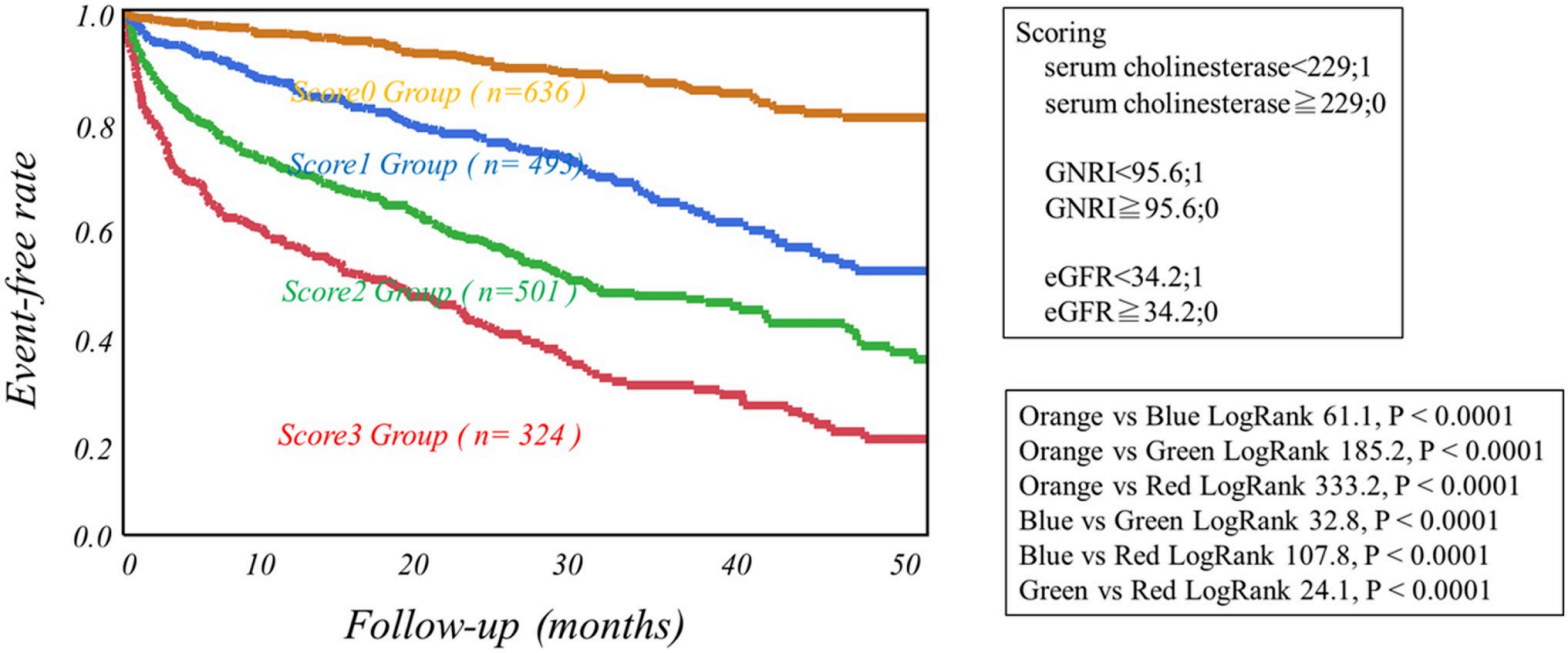
Kaplan-Meier event-free curves based on the accumulated number of the three prognostic variables including serum cholinesterase, Geriatric Nutritional Risk Index (GNRI) and estimated GFR.

Using cutoff values determined by ROC analysis, high-risk HF categories were clearly discriminated from low-risk categories as follows. Patients with serum cholinesterase less than 229 U/L, GNRI less than 95.6 or eGFR more than 34.2 ml/min/1.73m^2^ had significantly lower event-free rates than did the other patients (Fig 1). The combined use of eGFR or GNRI with serum cholinesterase more clearly discriminated patients with greater risks of CEs from others (Fig 2). Serum cholinesterase < 229 U/L, eGFR < 34.2 ml/min/1.73m^2^ and GNRI < 95.6 were incrementally related to increases in the rates of CEs, leading to the lowest CE-free curve when all of the three abnormalities were combined (Fig 3).

Furthermore, when heart failure patients in this study were analyzed in the subgroups of males and females, age (65 years old older or younger than 65 years), LVEF (reduced EF (EF<40%) or mid-range EF (EF≧40%)), etiology (ischemic or non-ischemic), and body mass index (BMI≦20, 20<BMI<25, BMI≧25), similar Kaplan-Meier event-free curves were obtained (S1–S5 Figs).

## Discussion

In this study serum cholinesterase and GNRI were shown to have critical roles for improvement in risk-stratification of systolic HF patients with renal dysfunction.

HF is a condition based on cardiac dysfunction, but non-cardiac factors including complications have a significant impact on the condition, which is one of the differences between HF and cancer. Against this backdrop, nutritional disorders, which are included in the non-cardiac factors, are attracting attention as a new target for therapeutic approaches for HF patients. A number of epidemiological studies have shown that nutritional impairment is a risk factor for the development of HF and for events after the onset of HF [12, 13]. Malnutrition, or cachexia, in HF is a concept that encompasses many factors including protein catabolism, lipolysis, and bone loss based on increased sympathetic nerve activity, increased inflammatory cytokines, and increased insulin resistance [14, 15].

In addition to the conventional cardiac function parameters and non-cardiac factors such as renal function, prognostic prediction with the parameter of nutritional status conducted in this study may contribute to the timing of determination of and the efficacy of nutritional management as non-pharmacological therapy called nutritional management in future treatment of HF.

Conventionally, since serum cholinesterase activity reflects liver function and nutritional status, a decrease in serum cholinesterase level has been regarded as an important surrogate parameter for liver dysfunction and malnutritional status.

Recently, it has been suggested that a negative spiral in which worsening of heart failure exacerbates nutritional deficits and the progression of nutritional deficits increases the severity of HF occurs after the onset of HF.

Recently, it has been reported that butyrylcholinesterase is related to muscle mass and strength and is a new biomarker to identify elderly people at risk for sarcopenia [16]. Moreover, serum cholinesterase has been attracting attention as an indicator of low nutrition in HF patients, and there are several reports on its potential to be a prognostic factor in HF patients [6, 17, 18].

Decreased renal function results in disruption of energy metabolism due to loss of homeostasis and metabolic function in the kidneys. In addition to the kidneys, metabolic abnormalities also occur in the liver, skeletal muscle, and adipose tissue, resulting in a high degree of systemic catabolism, making nutritional supplementation even more important [19].

Protein energy wasting (PEW), i.e., depletion of protein and energy sources, is a concept of malnutrition in chronic kidney disease. At present, there is no single parameter that enables diagnosis of PEW, and it is therefore diagnosed comprehensively by biochemical indices, weight loss, muscle loss, and decreased energy and protein intake [20].

Serum cholinesterase and GNRI may be potential biochemical indicators to assess PEW in chronic kidney disease patients with heart failure.

### Study limitations and future perspectives

This study was designed as an observational cohort study of HF patients with systolic dysfunction at a single center. A larger multicenter interventional study based on the present results could contribute to the development of better prevention and treatment strategies using appropriate indications for malnutrition in HF patients at high risk of death.

Moreover, assessment of right-sided heart function is becoming increasingly important because severe left-sided heart failure can result in bilateral heart failure, damaging both circulatory systems [21]. Severe right-sided heart failure can also damage the liver, which is responsible for nutritional management in the body due to congestion, contributing to malnutrition and sarcopenia. Therefore, additional evaluation of the parameters of right ventricular function and right ventricular pressure (TAPSE, PAP, and right atrial pressure) by echocardiography and Swan-Ganz catheterization may further stratify the risk in HF patients [22–24].

In addition, the prognostic impact of malnutrition on HF patients was not evaluated in this study by using the Mini Nutritional Assessment (MNA), Prognostic Nutrition Index (PNI) and Controlling Nutritional Status (CONUT), Subjective Global Assessment (SGA), and Nutritional Risk Screening (NRS) 2012 [25–29]. Another limitation is that arm circumference, which has been reported to improve the prognosis of heart failure patients when assessed together with BMI, was not measured [30].

There are two types of therapy for HF patients: pharmacological and non-pharmacological. Implantable cardioverter defibrillators and biventricular pacemakers have been attracting attention as non-pharmacological treatments, and the importance of nutritional management including diet and exercise rehabilitation should be further discussed.

## Conclusions

Low serum cholinesterase and low GNRI can independently and synergistically predict cardiac mortality risk in systolic HF patients with renal dysfunction.

## Supporting information

S1 Fig (A). eGFR, GNRI, and serum cholinesterase cut-off values determined by ROC analysis for the prediction of cardiac events by combination with the cut-off values of three parameters in the group of males. (B). eGFR, GNRI, and serum cholinesterase cut-off value determined by ROC analysis for the prediction of cardiac events by combination with the cut-off values of three parameters in the group of females.
(TIFF)

S2 Fig (A). eGFR, GNRI, and serum cholinesterase cut-off values determined by ROC analysis for the prediction of cardiac events by combination with the cut-off values of three parameters in the group of patients aged 65 years or less. (B). eGFR, GNRI and, serum cholinesterase cut-off values determined by ROC analysis for the prediction of cardiac events by combination with the cut-off values of three parameters in the group of patients aged more than 65years.
(TIFF)

S3 Fig (A). eGFR, GNRI, and serum cholinesterase cut-off values determined by ROC analysis for the prediction of cardiac events by combination with the cut-off values of three parameters in the group of patients with LVEF<40%. (B). eGFR, GNRI, and serum cholinesterase cut-off values determined by ROC analysis for the prediction of cardiac events by combination with the cut-off values three parameters in the group of patients with LVEF≧40%.
(TIFF)

S4 Fig (A). eGFR, GNRI, and serum cholinesterase cut-off values determined by ROC analysis for the prediction of cardiac events by combination with the cut-off values of three parameters in the ischemic etiology group. (B). eGFR, GNRI, and serum cholinesterase cut-off values determined by ROC analysis for the prediction of cardiac events by combination with the cut-off values of three parameters in the non-ischemic etiology group.
(TIFF)

S5 Fig (A). eGFR, GNRI, and serum cholinesterase cut-off values determined by ROC analysis for the prediction of cardiac events by combination with the cut-off values of three parameters in the group of patients with BMI≦20 (25% quantile). (B). eGFR, GNRI, and serum cholinesterase cut-off values determined by ROC analysis for the prediction of cardiac events by combination with the cut-off values of three parameters in the group with 20<BMI<25. (C). eGFR, GNRI, and serum cholinesterase cut-off values determined by ROC analysis for the prediction of cardiac events by combination with the cut-off values of three parameters in the group of patients with BMI≧25 (75% quantile).
(TIFF)
